# Endothelial cell senescence shapes T cell activity in late-stage of chronic obstructive pulmonary disease

**DOI:** 10.1038/s41420-026-03020-2

**Published:** 2026-03-20

**Authors:** Chae Min Lee, Jongchan Kim, Junhyup Song, Andrew Sehoon Kim, Sugyeong Jo, Nahee Hwang, Jae Woong Jeong, Minki Kim, Sung Jae Shin, Sungsoon Fang, Bo Kyung Yoon

**Affiliations:** 1 Graduate School of Medical Science, Brain Korea 21 Project, Yonsei University College of Medicine, Seoul, Republic of Korea; 2 Department of Biomedical Sciences, Gangnam Severance Hospital, Yonsei University College of Medicine, Seoul, Republic of Korea; 3 Department of Life Science and Biotechnology, Underwood Division, Underwood International College, Yonsei University, Seoul, Republic of Korea; 4 Department of Medicine, Yonsei University College of Medicine, Seoul, Republic of Korea; 5 Vanderbilt University, College of Arts and Science, Nashville, TN USA; 6 Institute for Immunology and Immunological Diseases, Yonsei University College of Medicine, Seoul, Republic of Korea; 7 Department of Microbiology and Immunology, Yonsei University College of Medicine, Seoul, Republic of Korea

**Keywords:** Chronic obstructive pulmonary disease, Prognostic markers

## Abstract

Chronic obstructive pulmonary disease (COPD) is a leading cause of death with few effective therapies. While clinical staging distinguishes mild to very severe disease, recent molecular and single-cell studies have revealed that progression involves distinct reprogramming of cellular and immune pathways rather than a simple linear escalation of inflammation. Yet, most studies have analyzed COPD without stratifying by stage, obscuring mechanisms specific to disease severity. To address this, we investigated serum proteomic profiles from the UK Biobank and applied machine learning to identify stage-specific protein signatures across COPD progression. Integration with single-cell and bulk transcriptomic datasets revealed that in severe COPD, endothelial cells exhibit a senescent phenotype characterized by elevated interleukin-6 (IL6) expression. Endothelial-derived IL6 correlated with reduced type 1 helper T cell (Th1) abundance and impaired interferon-γ signaling, indicating suppression of Th1-mediated immunity. These findings position endothelial senescence–driven IL6 signaling as a key pathogenic mechanism and potential therapeutic target in late-stage COPD.

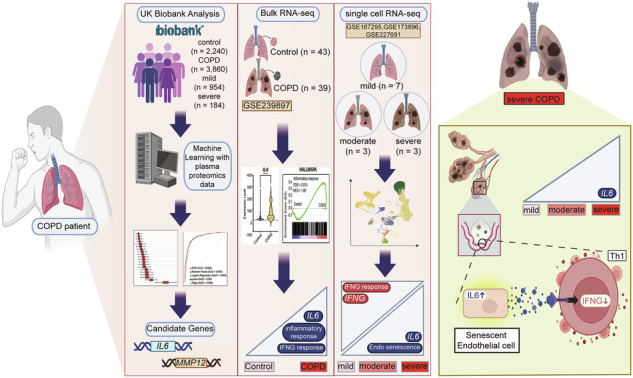

## Background

Chronic obstructive pulmonary disease (COPD) is the fourth leading cause of death worldwide, responsible for approximately 3.71 million deaths in 2021 [1, 2]. The incidence rate of COPD is expected to double by 2030 and is expected to cost the world economy 4.236 trillion dollars from 2020 to 2050 [3, 4]. Despite its global impact, existing therapeutic strategies—including bronchodilators, inhaled corticosteroids, phosphodiesterase-4 inhibitors, and Beta-2 Agonists—primarily focus on symptom management rather than reversing the underlying pathology [5]. These therapeutic strategies fall short of achieving durable disease control, with approximately 48.4% of COPD patients experiencing at least one severe exacerbation over a five-year period [6]. In addition, nearly 21% relapse within just 2 weeks following emergency department treatment for an acute exacerbation of COPD [7], underscoring the need for more effective on-spot and sustained therapeutics. Taken together, the status quo of COPD demands an urgent need for mechanistic research and innovative therapeutic strategies to mitigate its escalating global burden.

COPD pathogenesis is characterized by irreversible airflow limitation resulting from a combination of obstructive bronchiolitis and parenchymal destruction [8, 9]. Emerging research underscores the role of additional mechanisms that drive disease progression, which include persistent low-grade inflammation, oxidative stress upregulation, protease-antiprotease imbalance, and impaired tissue repair [10–13]. The advancement of COPD pathology is categorized into four stages—mild, moderate, severe, and very severe—based on the Global Initiative for Chronic Obstructive Lung Disease [14]. Although disease progression in COPD is associated with worsening prognosis and diminishing treatment options [15], inflammation does not correlate with disease progression in a simple linear manner. The common assumption that inflammatory burden increases proportionally with disease severity is increasingly challenged by evidence showing distinct immune microenvironments at each stage of COPD [16]. For instance, M1 and M2 phenotypes coexist within alveolar macrophages in late-stage COPD, while early-stage COPD features proliferating and monocyte-like macrophages [17, 18]. Such shifts in immune cell composition point to underlying biological processes that remodel the pulmonary immune microenvironment. Among the many factors influencing these evolving immune environments, cellular senescence is a particularly compelling mechanism that may shape COPD progression in a stage-dependent manner.

Cellular senescence is a state of stable cell-cycle arrest that plays a role in wound healing via growth factors such as PDGF-AA and cancer prevention by blocking uncontrolled cell division [19, 20]. However, continued research shows that senescence also exerts pathogenic effects by promoting chronic inflammation and immune dysregulation through the accumulation of senescent lung structural cells and their sustained senescence-associated secretory phenotype [21–24]. Among lung cell types that are particularly susceptible to senescence, endothelial cells are especially relevant in COPD because senescence-associated endothelial dysfunction compromises vascular barrier integrity, increases permeability, and contributes to vascular complications such as pulmonary hypertension [25, 26]. Mechanistically, endothelial senescence can promote fibrotic remodeling and reduce alveolar–capillary surface area and functions as an immunologic modulator through secretion of a diverse senescence-associated secretory phenotype (SASP) repertoire that can alter leukocyte recruitment, activation, and differentiation [27–29]. These signals foster chronic low-grade inflammation termed “inflammaging”, ultimately leading to immunosenescence [30, 31]. This altered immune landscape is evident in severe COPD, where senescence and aging-related signatures are markedly enriched compared to milder disease stages [32]. Although senescence has been well characterized with COPD, its stage-specific immunological consequences—particularly its interaction with cytokine signaling networks—remain poorly characterized. Clarifying how endothelial senescence shapes the immune microenvironment across COPD severity may provide critical insight into disease mechanisms and identify therapeutic opportunities that extend beyond symptom control.

In this study, we conducted a multi-layered analysis to investigate the immune landscape across different stages of COPD. Machine learning analysis applied to UK Biobank proteomics data identified candidate proteins associated with disease severity. Among the candidate proteins, IL6 emerged as a key biomarker enriched in severe COPD and linked to both disease severity and prognosis. Bulk and single-cell RNA (scRNA) sequencing analyses further revealed that IL6 expression was predominantly derived from endothelial cells exhibiting strong signatures of cellular senescence. The IL6 signaling targeted T cell, contributing to the distinct immunological profile of severe COPD characterized by depletion of interferon-gamma (IFN-γ) signaling and a reduced abundance of type 1 helper T cell (Th1). Collectively, these results reveal a distinct immunopathology in severe COPD, advocating for stage-specific treatment interventions. Overall, this study aims to clarify how senescence reshapes immunity at different stages of COPD and to uncover mechanisms that may guide stage-specific therapeutic strategies.

## Results

### UK Biobank blood proteome profiling uncovers key COPD biomarkers via machine learning analysis

Machine learning analysis was performed using UK Biobank blood proteome data to identify key proteomic biomarkers associated with COPD. Two independent models were developed: one comparing healthy controls and COPD patients, and the other comparing patients with mild versus severe COPD. Healthy controls (*n* = 23,421) were defined as individuals with no ICD-10 diagnoses, no self-reported cancer, and no self-reported non-cancer illness. COPD patients (*n* = 32,891) were identified based on at least one of the following criteria: an ICD-9 diagnosis of 492, 469, or 4969; an ICD-10 diagnosis of J43.0, or J44.0-J44.9; a self-reported diagnosis of COPD, chronic bronchitis, or emphysema; or a doctor-diagnosed record of COPD (Table 1). From this cohort, 2240 control participants and 3860 COPD patients had available plasma proteomic data. The demographic and clinical characteristic of the proteomic-available cohort is given in Table 2. This subset was randomly divided into a training set (control: *n* = 1568; COPD: *n* = 2702) and a test set (control: *n* = 672; COPD: *n* = 1158) for model development and evaluation.

**Table 1:** Categories of COPD-related patients in UK Biobank data.

Code	Diagnose
J41.0	Simple chronic bronchitis
J41.1	Mucopurulent chronic bronchitis
J41.8	Mixed simple and mucopurulent chronic bronchitis
J42	Unspecified chronic bronchitis
J43.0	MacLeod’s syndrome
J43.1	Panlobular emphysema
J43.2	Centrilobular emphysema
J43.8	Other emphysema
J43.9	Emphysema, unspecified
J44.0	Chronic obstructive pulmonary disease with acute lower respiratory infection
J44.1	Chronic obstructive pulmonary disease with acute exacerbation, unspecified
J44.8	Other specified chronic obstructive pulmonary disease
J44.9	Chronic obstructive pulmonary disease, unspecified

The International Classification of Diseases, 10th revision codes used to filter patients in the UK Biobank dataset to identify trends in a large number of patients.

**Table 2:** Demographic and clinical characteristics of each UK Biobank cohort.

Characteristics	Control (*n* = 2240)	COPD (*n* = 3860)	*p*-value
**Sex**			
Female	1164 (52.0)	1817 (47.1)	2.54e-4
Male	1076 (48.0)	2043 (52.9)	
**Ethnicity**			
British	1855 (82.8)	3432 (88.9)	–
Other White	162	290	
Asian	72	50	
African	75	8	
Others*	76	80	
**Ever smoked**			
Yes	1199 (53.5)	3134 (81.2)	<2.2e-16
No	1041 (46.5)	726 (18.8)	
**Alcohol consumption: current status**			
Yes	2081 (92.9)	3376 (87.5)	3.42e-11
No	159 (7.1)	484 (12.5)	
Age, y	68 (62–74)	79 (74–83)	<2.2e-16
Weight, kg	73.9 (64.2–83.8)	78.8 (67.8–90.5)	<2.2e-16
BMI, kg/m²	25.5 (23.2–28.0)	27.8 (24.7–31.4)	<2.2e-16
FEV1% (Forced Expiratory Volume in 1 s), L	2.98 (2.50–3.60)	2.19 (1.72–2.73)	<2.2e-16
PEF (Peak Expiratory Flow), L/min	405 (327–508)	313 (235–397)	<2.2e-16

^*^Ethnicity – Others: mixed background, Caribbean, Do not know, Prefer not to answer, etc.

Categorical Variables → (Percentage); *p*-value (Chisq).

Continuous Variables → Median (interquartile range); *p*-value (Mann–Whitney U).

Comparison between healthy controls (NOR, *n* = 2240) and individuals with COPD (*n* = 3860) across sex, ethnicity, smoking status, alcohol use, age, weight, BMI, and lung function is shown. Data are presented as counts (percentages) for categorical variables and as median (interquartile range) for continuous variables. *P* values were calculated using the Chi-square test for categorical variables and the Mann–Whitney *U* test for continuous variables. Ethnicity categories include British, Other White, Asian, African, and Others (including mixed background, Caribbean, unknown, and undisclosed).

**Fig. 1:**
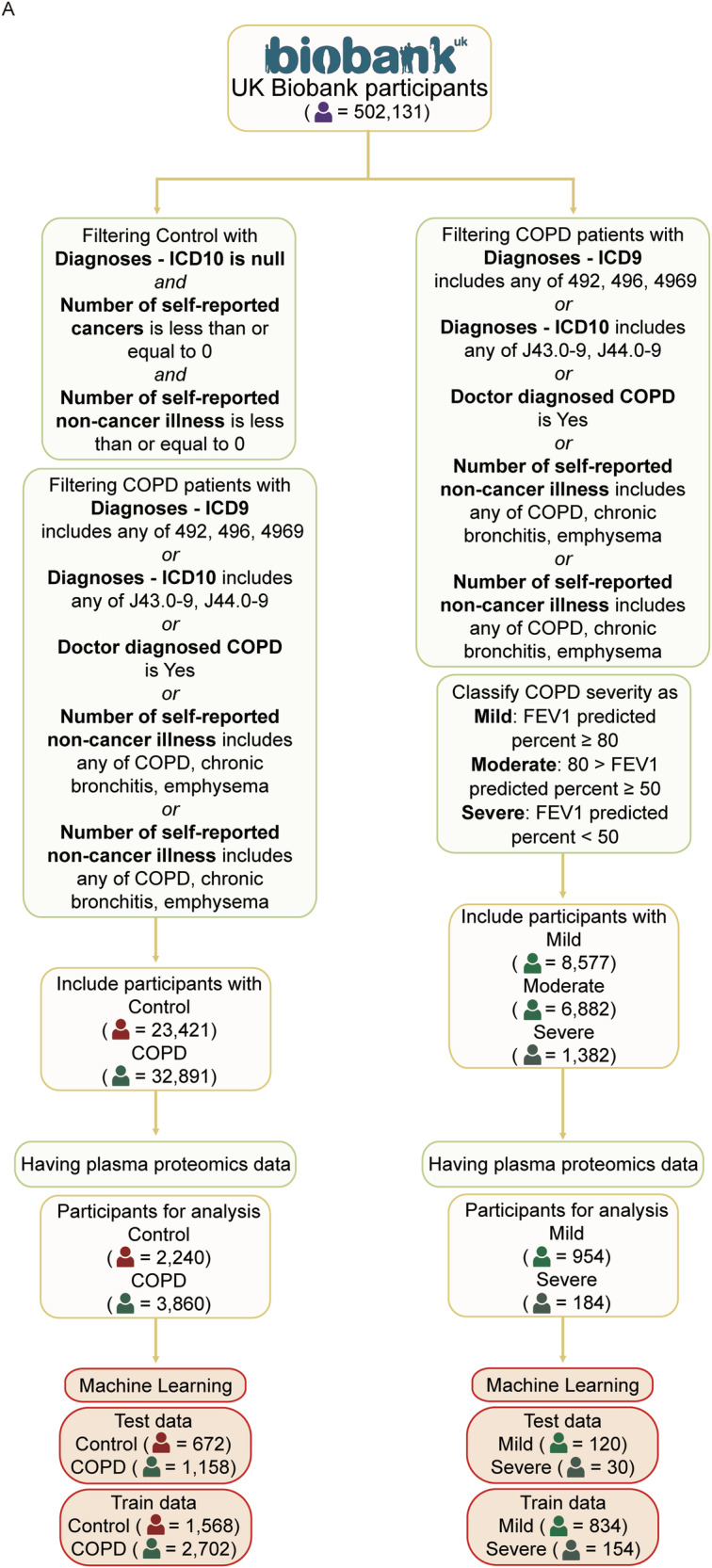
Schematic overview of participant selection from the UK Biobank for proteomics-based machine learning analysis. A Flowchart illustrating the step-by-step selection process of participants from the UK Biobank dataset for downstream machine learning analysis using plasma proteomic data. Two distinct machine learning pipelines are implemented: (1) classification of COPD patients versus normal healthy controls, and (2) stratification of COPD patients into mild, moderate, and severe subgroups based on the FEV1% predicted percentage. These selection and stratification processes enable the identification of candidate proteins associated with both disease presence and severity. ICD International Classification of Diseases, FEV1% Forced Expiratory Volume in one second, COPD Chronic Obstructive Pulmonary Disease.

COPD severity was further stratified based on percent predicted forced expiratory volume in one second (FEV1%). Patients with an FEV1% ≥ 80 were classified as mild, and those with an FEV1% < 50 as severe. According to these criteria, 8577 mild, 6882 moderate, and 1382 severe COPD patients were identified. Of these, 954 mild and 184 severe patients had available plasma proteomic data. This group was further split into a training set (mild; *n* = 834; severe: *n* = 154) and a test set (mild; *n* = 120; severe: *n* = 30) for downstream analysis (Fig. 1A).

### IL6 and MMP12 in blood are strongly associated with COPD severity and survival

**Fig. 2:**
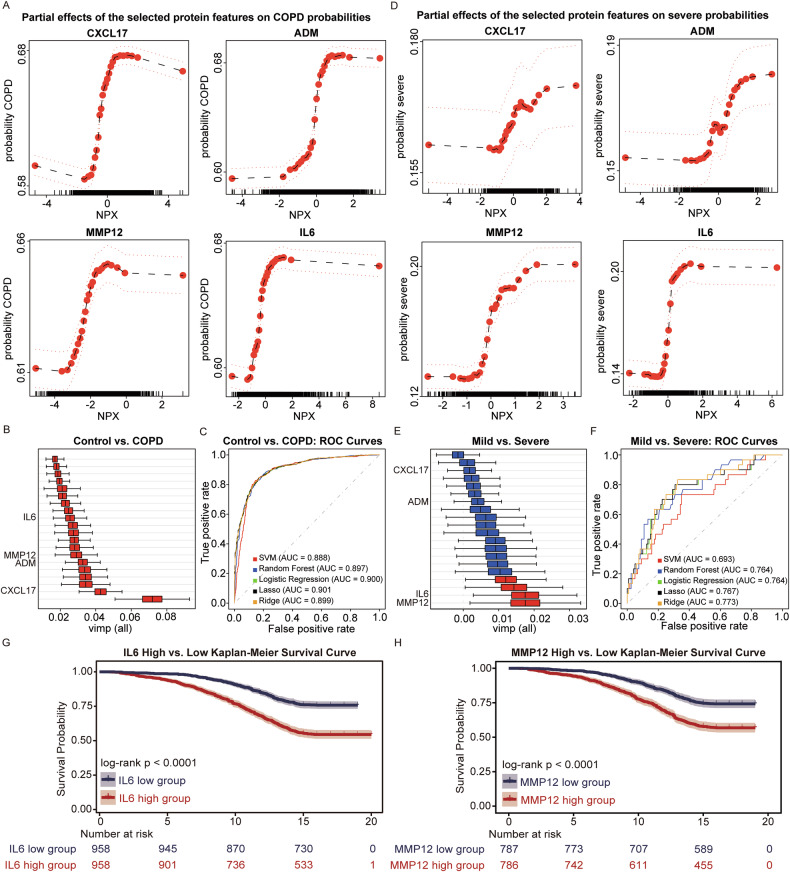
Identification of key proteins associated with COPD diagnosis and severity using machine learning. Partial dependence plots showing the effects of four overlapping proteins (CXCL17, ADM, MMP12, and IL6) on predicted probabilities of COPD diagnosis (**A**) and COPD severity (**D**). These proteins were consistently selected as key features in both classification tasks. Variable importance plot displaying the top 20 proteins contributing to the classification of COPD patients versus normal healthy controls (**B**) and severe COPD versus mild COPD patients (**E**). Red bars indicate proteins with lower CI boundaries above 0, while blue bars represent those with lower CI boundaries below 0. MMP12 and IL6 are highlighted as common and robust predictors in both comparisons. ROC curves comparing the classification performance of five machine learning models in distinguishing normal from COPD (**C**) and mild from severe COPD (**F**). AUC values greater than 0.7 indicate acceptable performance, and values above 0.8 reflect good classification accuracy. Based on the results from (**A**–**F**), IL6 and MMP12 were considered as key proteins associated with both COPD status and severity. COPD patients in the UK Biobank cohort were stratified into high and low expression groups for IL6 (**G**) and MMP12 (**H**) and Kaplan–Meier survival analyses were performed. In both cases, patients in the high-expression group exhibited significantly lower survival probabilities compared to those in the low-expression group. NPX Normalized protein expression, FDR False discovery rate, SVM Support vector machine, AUC Area under the curve, LASSO Least absolute shrinkage and selection operator, vimp Variable importance, CI Confidence interval, ROC Receiver operating characteristic.

Machine learning was trained using the filtered UK Biobank dataset through a previously validated pipeline [33]. Partial dependence plots revealed four common protein markers—CXCL17, ADM, MMP12, and IL6—consistently associated with both COPD onset (Fig. 2A) and progression (Fig. 2D). For all four proteins, an increase in normalized protein expression (NPX) corresponded with a higher probability of COPD diagnosis or classification into the severe group. These findings suggest that elevated expression of these proteins may contribute to both disease onset and progression.

To further interpret model outputs, variable importance was computed and ranked for the top 20 protein features in each classification task (Fig. 2B, E). In the healthy control versus COPD comparison, most features exhibited strong importance scores, whereas in the mild versus severe analysis, only four proteins showed confidence intervals with upper bounds clearly above zero, again highlighting IL6 and MMP12 as robust markers.

Model performance was evaluated using receiver operating characteristic (ROC) curves across five machine learning models: support vector machine (SVM), random forest, logistic regression, least absolute shrinkage and selection operator (LASSO) and ridge regression (Fig. 2C, F). The control versus COPD task demonstrated high classification accuracy, with area under the ROC curve (AUC) values ranging from 0.888 to 0.901. The mild versus severe classification also showed consistent performance, with slightly lower AUC values between 0.693 and 0.773. These results confirm the stability and generalizability of the models across different algorithms.

Given the consistent appearance of IL6 and MMP12 among the top-ranked features in both classification settings, COPD patients were stratified into high- and low-expression groups for each protein based on the mean NPX values. Kaplan–Meier survival analysis revealed significantly reduced survival probabilities in the high-expression groups for both IL6 and MMP12 (Fig. 2G, H), suggesting that elevated levels of these proteins are associated not only with disease presence and severity but also with poor clinical outcomes. Multivariable logistic regression analysis was conducted to further assess the associations between IL6, MMP12, and other variables. The model was adjusted for age, sex, body mass index (BMI), and smoking status. Both IL6 and MMP12 were found to be independently associated with COPD. Increased levels of IL6 (OR = 1.79, 95% CI: 1.63–1.97) and MMP12 (OR = 1.89 and 95% CI: 1.72–2.07) were significantly correlated with a higher likelihood of COPD diagnosis. These findings support the potential role of IL6 and MMP12 as distinct biomarkers for COPD and its progression. The result is described in Table 3.

**Table 3:** Multivariable logistic regression analysis of factors associated with COPD.

Value	OR	95% CI	*P*-value
**IL6**	1.79	1.63–1.97	<0.001
**MMP12**	1.89	1.72–2.07	<0.001
**Age**	1.12	1.11–1.14	<0.001
**BMI**	1.08	1.06–1.10	<0.001
Ever smoked*	2.97	2.55–3.47	<0.001
**Sex**	0.93	0.81–1.07	0.32

^*^Ever smoker was defined as participants who reported current or former smoking. Individuals with missing or unclear smoking information were excluded from the analysis.

### Transcriptomic profiling of the lung reveals upregulation of IL6 signaling in severe COPD patients

**Fig. 3:**
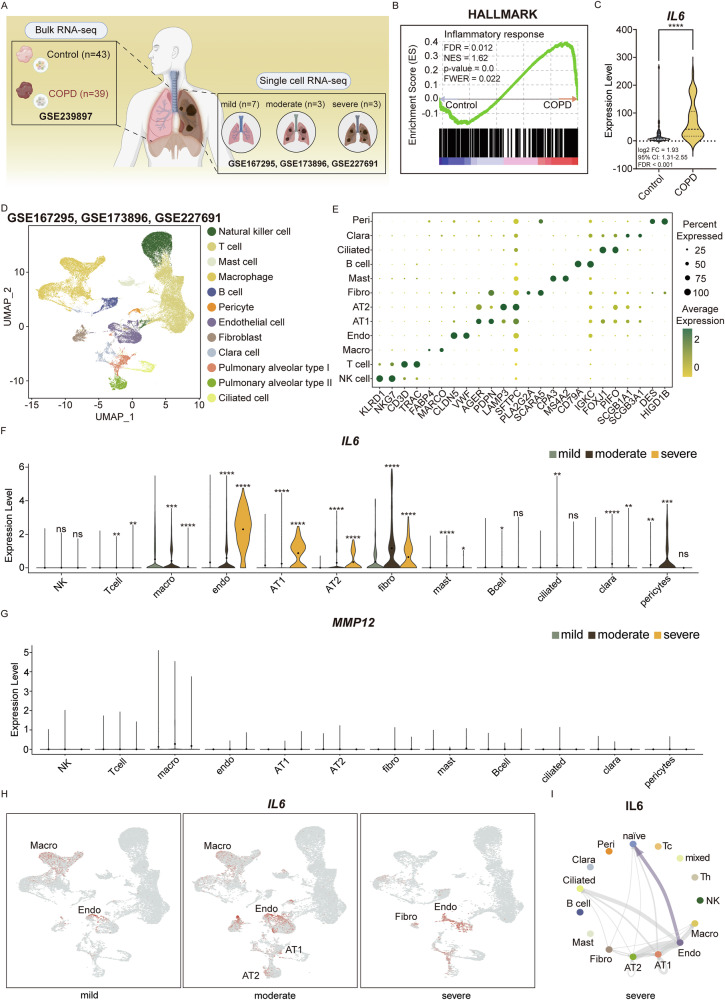
Bulk and scRNA sequence analysis reveal *IL6* expression dynamics and inflammatory signaling in COPD lungs. **A** Overview of bulk and scRNA sequencing datasets used in the analysis. Bulk RNA sequencing data from normal (*n* = 43) and COPD (*n* = 39) lung tissues were obtained from GSE239897. ScRNA sequencing datasets from mild (*n* = 7), moderate (*n* = 3), and severe (*n* = 3) patients were retrieved from GSE167295, GSE173886, and GSE227651. The figure was prepared using BioRender, with publication and licensing rights confirmed under Agreement No. SS29EUGDP4. **B** Enrichment plots showing elevated enrichment scores for the Inflammatory response gene set in COPD patients compared to normal individuals in the Hallmark database. Enrichment of Inflammatory response was observed with an FDR of 0.015 and an NES of 1.60, indicating a statistically significant upregulation of Inflammatory response in COPD patients compared to healthy controls. **C** Violin plot demonstrating significantly increased *IL6* expression in COPD lungs compared to normal controls in bulk RNA sequencing data, while *MMP12* does not show a statistically significant difference. **D** UMAP of the integrated COPD scRNA sequencing dataset, comprising 55,154 cells clustered into 12 distinct cell types. **E** Dot plot revealing the expression of cell type-specific marker genes used to annotate the 12 clusters identified in dot size indicates the percentage of cells expressing each gene, and color intensity reflects the average expression level. **F** Violin plot illustrating the expression levels of *IL6* across cell types and COPD severity stages. *IL6* expression is notably increased in endothelial cells during the severe stage. **G** Violin plot visualizing the expression levels of *MMP12* across cell types and COPD severity stage. *MMP12* expression remains low and non-distinct. **H** Feature plots visualizing *IL6* expression across UMAP clusters for mild, moderate, and severe COPD. Despite a reduction in the endothelial cell population in severe COPD, *IL6* expression remains highly enriched in this cell type **I** Circle plot illustrating inferred IL6-mediated signaling in severe COPD. IL6 signaling is primarily received by naïve T cells. (ns=not significant, * *p* < 0.1, ** *p* < 0.01, *** *p* < 0.001, **** *p* < 0.0001); scRNA single-cell RNA, COPD Chronic Obstructive Pulmonary Disease, FDR False discovery rate, NES Normalized enrichment score, FWER Family-wise type 1 error rate, FC Fold Change, UMAP Uniform manifold approximation and projection, NK cell Natural killer cell, Mast Mast cell, Macro Macrophage, Peri Pericyte, Endo Endothelial cell, Fibro Fibroblast, Clara Clara cell, Pulmonary alveolar type I (AT1), Pulmonary alveolar type II (AT2), Ciliated Ciliated cell, naïve naïve T cells, Tc Cytotoxic T cell, Th Helper T cell, mixed T cell mixed.

Transcriptomic analysis was employed to elucidate the gene-level regulation and cellular interactions of IL6 and MMP12 identified through prior proteomic profiling. Bulk RNA sequencing was analyzed by comparing lung tissue from 43 healthy individuals and 39 patients with COPD from the GSE239897 dataset. In parallel, scRNA sequencing was analyzed on lung tissue samples from 7 mild, 7 moderate, and 3 severe COPD patients from the GSE227691, GSE173896, and GSE167295 dataset (Fig. 3A and Tables 4–7). Analysis using the Hallmark Inflammatory response gene set revealed high enrichment scores for the inflammatory response pathway in COPD patients (Fig. 3B). Among key proinflammatory mediators, *IL6* was significantly upregulated in COPD compared to the healthy control (Fig. 3C). ScRNA sequencing was employed to perform stage-specific analysis of COPD, resulting in the identification of 12 discrete cell-type clusters (Figs. 3D and S1A). These clusters were classified based on marker genes as follows: natural killer (NK) cells (*KLRD1*, *NKG7*), T cells (*CD3D*, *TRAC*), macrophages (macro/ *FABP4*, *MARCO*), endothelial cells (endo/ *CLDN5*, *VWF*), pulmonary alveolar type I cells (AT1/ *AGER*, *PDPN*), pulmonary alveolar type II cells (AT2/ *LAMP3*, *SFTPC*), fibroblasts (fibro/ *PLA2G2A*, *SCARA5*), mast cells (*CPA3*, *MS4A2*), B cells (*CD79A*, *IGKC*), ciliated cells (*FOXJ1*, *PIFO*), clara cells (*SCGB1A1*, *SCGB3A1*), and pericytes (*DES, HIGB1B*) [34–38] (Figs. 3E and S1B*)*. Single-cell transcriptomic profiling revealed a divergence in gene expression patterns between the two UK Biobank-derived markers, MMP12 and IL6. *MMP12* expression remained unaltered across disease states, whereas *IL6* was markedly upregulated in severe COPD, particularly within endothelial cells, AT1 and AT2. (Fig. 3F, G). Within the severe COPD context, endothelial cells exhibited both the highest proportion of *IL6*⁺ cells and the highest mean *IL6* expression among all annotated cell types (Fig. S1C, D). Analysis of two independent cohorts—the COPD Cell Atlas (COPD *n* = 15; control *n* = 17) and GSE302339 (early-stage COPD *n* = 24; late-stage COPD *n* = 13)—consistently demonstrated endothelial-specific *IL6* upregulation as a COPD-associated feature, with endothelial cells from late-stage COPD patients exhibiting higher *IL6* expression than early-stage COPD endothelial cells [39, 40] (Fig. S1E, F). High *IL6* expression in severe COPD was associated with a high IL6 signaling toward naïve T cells in severe COPD from endothelial cells, implicating the IL6-mediated pathway as a potential immune regulator of severe COPD pathogenesis (Figs. 3I and S1G).

**Table 4:** Information and classification of COPD scRNA sequencing data.

	Dataset	Labeling	Identification	FEV1%	Sex	Age
**mild**	GSE227691	GSM7105665	Mild 1	97.3	Male	52
	GSE227691	GSM7105666	Mild 2	112.7	Male	76
	GSE227691	GSM7105667	Mild 3	92.9	Male	67
	GSE227691	GSM7105668	Mild 4	109.4	Male	61
	GSE173896	JK05	Mild 5	102	Female	70 s
	GSE173896	JK07	Mild 6	82.6	Male	80 s
	GSE173896	JK08	Mild 7	88.7	Male	80 s
**moderate**	GSE227691	GSM7105669	Moderate 1	78.7	Male	70
	GSE227691	GSM7105670	Moderate 2	62.6	Male	80
	GSE227691	GSM7105671	Moderate 3	70.5	Male	61
	GSE227691	GSM7105672	Moderate 4	76.8	Male	78
	GSE173896	JK02	Moderate 5	79.6	Male	60 s
	GSE173896	JK09	Moderate 6	77.1	Male	70 s
	GSE173896	JK10	Moderate 7	74.6	Male	80 s
**severe**	GSE167295	100998	Severe 1	33	Female	65
**very severe**	GSE167295	100999	Severe 2	22	Female	62
	GSE167295	101000	Severe 3	21	Female	61

Detailed information is provided for a total of 17 COPD patients from GSE227691, 173896, and 167295. Forced expiratory volume in one second (FEV1%).

**Table 5:** Mild COPD scRNA sequencing data quality control.

	Mild 1	Mild 2	Mild 3	Mild 4	Mild 5	Mild 6	Mild 7
**Labeling**	GSM7105665	GSM7105666	GSM7105667	GSM7105668	JK05	JK07	JK08
**nFeature**	>100 <3000	>100 <3000	>100 <3200	>100 <3200	>0 <7500	>0 <9000	>0 <7000
**percent.mt**	<10	<8	<7	<7	<10	<10	<10
**Min. Cell**	3	3	3	3	3	3	3
**Min. Feature**	200	200	200	200	200	200	200
**PCA_npcs**	20	20	20	20	20	20	20
**UMAP_Dims**	1:10	1:10	1:10	1:10	1:10	1:10	1:10
**Doublet_pN**	0.25	0.25	0.25	0.25	0.25	0.25	0.25
**Doublet_pK**	0.09	0.09	0.09	0.09	0.09	0.09	0.09
**Doublet_PCs**	1:10	1:10	1:10	1:10	1:10	1:10	1:10

Quality Control information of mild COPD that was used in the downstream analysis.

**Table 6:** Moderate COPD scRNA sequencing data quality control.

	Moderate 1	Moderate 2	Moderate 3	Moderate 4	Moderate 5	Moderate 6	Moderate 7
**Labeling**	GSM7105669	GSM7105670	GSM7105671	GSM7105672	JK02	JK09	JK10
**nFeature**	>100 <2600	>100 <3200	>100 <2000	>100 <2200	>0 <6250	>0 <7500	>0 <7500
**percent.mt**	<10	<8	<6	<9	<10	<10	<10
**Min. Cell**	3	3	3	3	3	3	3
**Min. Feature**	200	200	200	200	200	200	200
**PCA_npcs**	20	20	20	20	20	20	20
**UMAP_Dims**	1:10	1:10	1:10	1:10	1:10	1:10	1:10
**Doublet_pN**	0.25	0.25	0.25	0.25	0.25	0.25	0.25
**Doublet_pK**	0.09	0.09	0.09	0.09	0.09	0.09	0.09
**Doublet_PCs**	1:10	1:10	1:10	1:10	1:10	1:10	1:10

Quality Control information of moderate COPD was used in the downstream analysis.

**Table 7:** Quality Control of scRNA sequencing data of severe COPD.

	Severe 1	Very severe 1	Very severe 2
**Labeling**	100998	100999	101000
**nFeature**	>0 < 4500	>0 < 4500	>0 < 4100
**percent.mt**	<10	<10	<10
**Min. Cell**	3	3	3
**Min. Feature**	200	200	200
**PCA_npcs**	20	20	20
**UMAP_Dims**	1:10	1:10	1:10
**Doublet_pN**	0.25	0.25	0.25
**Doublet_pK**	0.09	0.09	0.09
**Doublet_PCs**	1:10	1:10	1:10

Quality Control information of severe COPD that was used in the downstream analysis.

### Senescent endothelial cell is the major *IL6*–producing cell population in severe COPD

**Fig. 4:**
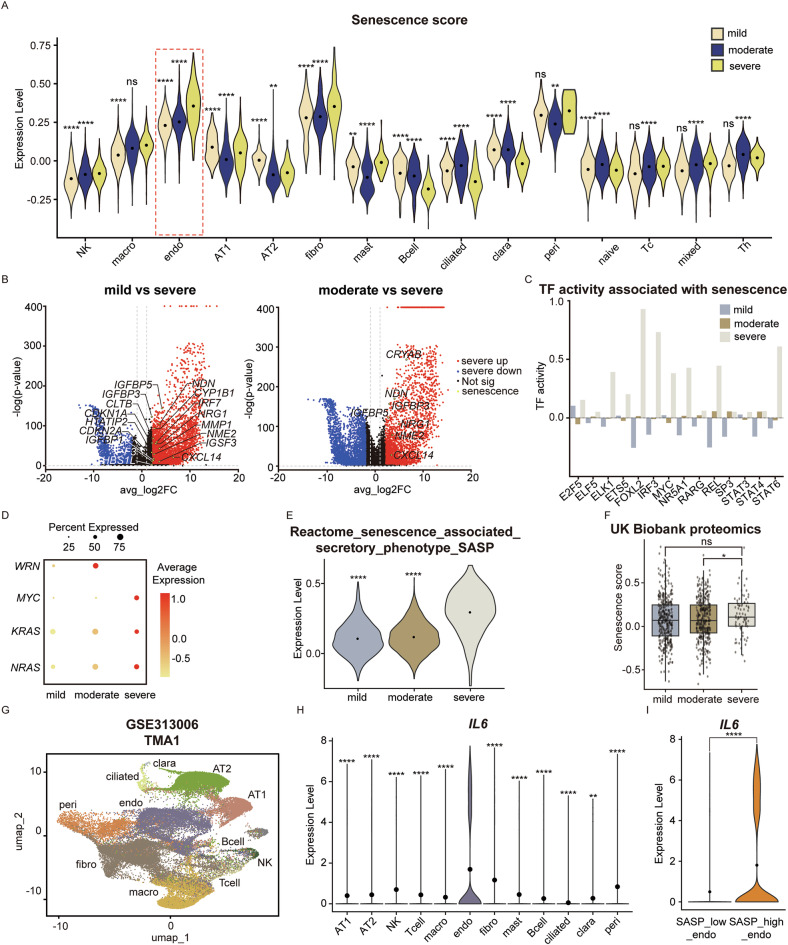
*IL6*-secreting endothelial cells in severe COPD is senescent endothelial cells. **A** Violin plot of senescence score according to mild, moderate, and severe COPD illustrating the increase of senescence in endothelial cells in severe COPD. **B** Volcano plot presenting the DEGs between mild and severe, and moderate and severe COPD endothelial cells. Both comparisons show upregulation of senescence-related genes in severe COPD endothelial cells. **C** Bar plot showing upregulation of senescence-related transcription factors in severe COPD endothelial cells compared to mild and moderate COPD, confirming cellular senescence in severe COPD endothelial cells. **D** Dot plot showing the gene expression of *WRN, MYC*, *KRAS*, and *NRAS,* which are proteins related to regulating senescence. Absence of *WRN* and the presence of *MYC*, *KRAS*, and *NRAS* show endothelial cell senescence in severe COPD. **E** Violin plot illustrating the expression levels of SASP across mild, moderate, and severe COPD. A significant increase of SASP is observed in severe COPD compared to mild and moderate COPD in endothelial cells. **F** Box plot of UK Biobank proteomic data showing an increase of NPX regarding senescence in severe COPD patients compared to mild and moderate COPD. (ns=not significant * *p* < 0.1, ** *p* < 0.01, **** *p* < 0.0001.) **G** Dim plot illustrating cell-type annotations in the COPD_TMA1 sample derived from the spatial transcriptomic dataset GSE313006. **H** Violin plot showing significantly elevated IL6 expression in endothelial cells compared with other cell types in COPD_TMA1 (** *p* < 0.01, **** *p* < 0.0001). **I** Violin plot showing SASP-high endothelial cells exhibit an upregulated IL6 expression compared to SASP-low endothelial cells in COPD_TMA1 (**** *p* < 0.0001); DEGs Differentially expressed genes, NK cell Natural killer cell, Mast Mast cell, Macro Macrophage, B cell B cell, Peri Pericyte, Endo Endothelial cell, Fibro Fibroblast, Clara Clara cell, AT1 Pulmonary alveolar type I, AT2 Pulmonary alveolar type II, Ciliated Ciliated cell, naïve naïve T cells, Tc Cytotoxic T cell, Th Helper T cell, mixed T cell mixed, TF transcription factor, avg_log2FC average log2 fold change, NPX Normalized protein expression, SASP Senescence-associated secretory phenotype, TMA Tissue Microarry.

Given that endothelial cells are not an immune cell in origin, the marked upregulation of *IL6* in severe COPD endothelial cells warrants further investigation. Previous studies have shown that chronic endothelial cell inflammation can induce cellular senescence, leading to a high *IL6* expression as part of the SASP [41]. This suggests endothelial cell senescence as a potential upstream driver of IL6 upregulation within severe COPD patients. Thus, we examined senescence-related signatures across lung cell types (Fig. 4A and Table 8). Endothelial cells exhibited an increase in senescence signature in severe COPD, with supported converging lines of evidence. Firstly, a marked upregulation of canonical senescence-related genes—*CDKN1A, CXCL8, CXCL1, CCL2, ICAM1, and IGFBP7*—in severe COPD endothelial cells was observed compared to both mild and moderate COPD [42–45] (Figs. 4B and S2A). Secondly, transcription factor activity linked to senescence programs such as E2F5 and REL showed significantly increased activity in severe COPD, reinforcing the activation of senescence-associated transcriptional networks [46, 47] (Fig. 4C). Thirdly, analysis of endothelial cell gene expression revealed a pattern of *MYC, KRAS*, and *NRAS* upregulation in the absence of *WRN* expression (Fig. 4D). *WRN* encodes a RecQ helicase essential for genome stability through its regulation of DNA repair pathways, including homologous recombination, base excision repair and nonhomologous end joining. Studies have reported that *WRN* deficiency in the context of *MYC* upregulation leads to the induction of cellular senescence, further corroborating the presence of senescence in severe COPD endothelial cells [48]. Lastly, SASP expression dramatically increased in severe COPD endothelial cells which affirmed a robust senescence phenotype in severe COPD endothelial cells (Fig. 4E). This signature was further mirrored in the proteomics data from the UK Biobank which revealed a significant elevation of senescence-related protein signatures in the blood of individuals with severe COPD (Fig. 4F). To investigate the mechanism of endothelial cell senescence in severe COPD, TGF-β signaling- a canonical senescence inducer- was investigated [49]. Surprisingly, this pathway was downregulated, suggesting that endothelial cell senescence in severe COPD may arise through a TGF-β–independent mechanism (Fig. S2B).

**Table 8:** Gene list of Senescence_score.

**Senescence_score**	*ALDH1A3*	*CCND1*	*CD44*	*CDC26*	*CDC27*
	*CDK2*	*CDK4*	*CDK6*	*CDKN1A*	*CDKN1B*
	*CDKN1C*	*CDKN2A*	*CDKN2B*	*CDKN2C*	*CDKN2D*
	*CITED2*	*CLTB*	*COL1A2*	*CREG1*	*CRYAB*
	*CXCL14*	*CYP1B1*	*EIF2S2*	*ESM1*	*F3*
	*FILIP1L*	*FN1*	*GSN*	*GUK1*	*HBS1L*
	*HPS5*	*HSPA2*	*HTATIP2*	*IFI16*	*IFNG*
	*IGFBP1*	*IGFBP2*	*IGFBP3*	*IGFBP4*	*IGFBP5*
	*IGFBP6*	*IGFBP7*	*IGSF3*	*ING1*	*IRF5*
	*IRF7*	*ISG15*	*MAP1LC3B*	*MAP2K3*	*MDM2*
	*MMP1*	*NDN*	*NME2*	*NRG1*	*OPTN*
	*PEA15*	*RAB13*	*RAB31*	*RAB5B*	*RABGGTA*
	*RAC1*	*RBL2*	*RGL2*	*RHOB*	*RRAS*
	*S100A11*	*SERPINB2*	*SERPINE1*	*SMPD1*	*SMURF2*
	*SOD1*	*SPARC*	*STAT1*	*TES*	*TFAP2A*
	*TGFB1I1*	*THBS1*	*TNFAIP2*	*TNFAIP3*	*TP53*
	*TSPYL5*	*VIM*			

The senescence-score gene set was derived from Fridman_Senescence_Up, with unexpressed genes omitted from the gene set.

Consistent with the proposed mechanism, we performed in situ validation of IL6 upregulation in senescent endothelial cells using four independent COPD tissue microarray (TMA) samples. Across all 4 TMAs, we consistently observed a spatial colocalization of IL6 with CDKN1A, a well-established marker of cellular senescence, supporting the presence of senescence-associated IL6 expression [42] (Fig. S3A–D). For downstream analyses, cells within the TMAs were annotated into AT1, AT2, NK, T cell, macrophage, endothelial, fibroblast, mast, B cell, ciliated, and clara cell populations (Figs. 4G and S3E). Cell-type–resolved analysis revealed that endothelial cells were the predominant source of IL6 expression across all TMAs (Figs. 4H and S3F). To assess whether endothelial *IL6* upregulation was specifically associated with cellular senescence, we curated a senescence gene set based on REACTOME_CELLULAR_SENESCENCE, excluding genes with no detectable expression, genes that negatively regulate senescence, and IL6 itself. IL6 was intentionally excluded to ensure senescence-based cell stratification independent of IL6, enabling an unbiased evaluation of IL6 expression across senescence-defined cell populations. The final “senescence” gene set comprised *IL1A, CDKN1A, CDKN2A, JUN, NFKB1*, and *STAT3* [42, 50, 51] (Table 9). Using this gene set as a reference, endothelial cells were stratified into SASP-high and SASP-low populations. SASP-high endothelial cells exhibited a marked increase in IL6 expression compared with senescence-low endothelial cells in all 4 TMAs (Figs. 4I and S3G). Collectively, these findings demonstrate that IL6 upregulation in pulmonary endothelial cells during COPD is closely linked to cellular senescence, supporting an endothelial senescence-driven inflammatory mechanism in COPD pathogenesis.

**Table 9:** Gene list of “Senescence” gene set.

**Senescence**	*IL1A*	*JUN*
	*CDKN1A*	*NFKB1*
	*CDKN2A*	*STAT3*

The senescence gene set was derived from REACTOME_CELLULAR_SENESCENCE, excluding unexpressed genes and genes that negatively regulate senescence, as well as IL6. Although IL6 is a recognized marker of senescence, it was intentionally omitted to enable senescence-based cell stratification independent of IL6, allowing unbiased evaluation of IL6 expression across senescence-defined cell populations.

### T cells in severe COPD exhibit diminished IFN-γ production and response driven by IL6 signaling

**Fig. 5:**
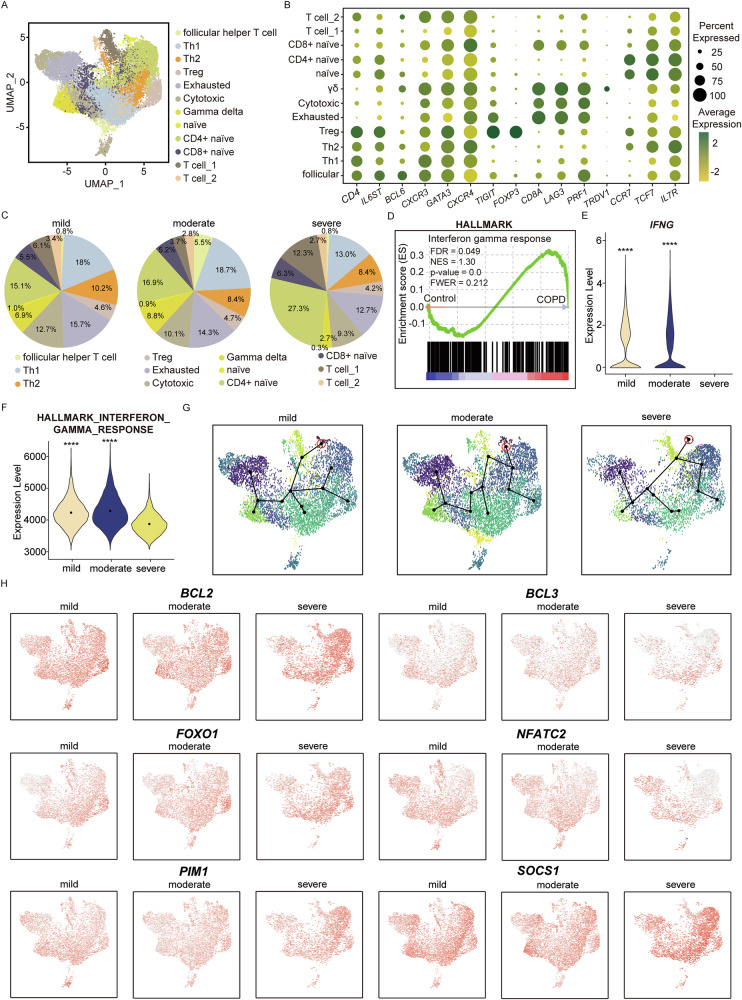
T cell-specific transcriptional signatures are selectively altered in severe COPD. **A** UMAP showing 12 clusters of subsets of T cells. **B** Dot plot illustration showing the key gene markers for each cluster of T cells. Dot size indicates the percentage of cells expressing each gene, and color intensity reflects average expression level. **C** Pie chart showing the population proportion of the subset of T cells across mild, moderate, and severe COPD. While CD4+naïve T cells are increased in severe COPD, Th1 is decreased in severe COPD compared to mild, moderate, and severe COPD. **D** Gene set enrichment of Hallmark Interferon Gamma Response with bulk RNA sequencing data showing that the COPD patient has a higher Interferon gamma response compared to normal control. Enrichment of the Interferon gamma response was observed with an FDR of 0.044 and an NES of 1.33, indicating a statistically significant upregulation of Interferon gamma response in COPD patients compared to healthy control. **E** Violin plot shows that at the gene level, *IFN-γ* is highly expressed in mild and moderate COPD but not in severe COPD. **F** Violin plot showing that when analyzed in a stage-specific manner with Hallmark Interferon gamma response, severe COPD has a decreased response of interferon gamma compared to mild and moderate COPD. **G** Pseudotime illustrates the differentiation of T cells across mild, moderate, and severe COPD, showing a unique differentiation path for severe COPD. **H** Feature plot showing key genes in IL6-JAK-STAT3-signaling upregulated in severe COPD compared to mild and moderate COPD, which confirms the effect of *IL6* in T cells in severe COPD. (**** *p* < 0.0001); follicular follicular helper T cell, Th1 type 1 Helper T cells, Th2 type 2 Helper T cells, Treg regulatory T cells, Exhausted exhausted T cells, Cytotoxic cytotoxic T cells, γδ gamma delta T cells, naïve naïve T cells, CD4+ naïve CD4+ naïve T cells, CD8+ naïve CD8+ naïve T cells, FDR false discovery rate, NES normalized enrichment score, FWER Family-wise type 1 error rate.

Given the IL6 signal emitted from the senescent endothelial cells in severe COPD targeting naïve T cells, scRNA sequencing data were used to further investigate T cells across COPD stages. Analysis of T cells from 7 mild, 7 moderate, and 3 severe COPD patients yielded a total of 18,601cells which were categorized into 12 clusters (Fig. 5A). Cluster identities were assigned based on the expression profiles of established marker genes: follicular helper T (follicular/*IL6ST*, *BCL6, CD4*), type 1 Helper T cell (Th1/*CXCR3*, *CD4*), type 2 Helper T cell (Th2/ *GATA3*, *CXCR4, CD4*), Regulatory T cell (Treg, *FOXP3*, *TIGIT*), Exhausted T cells (exhausted/ *TIGIT, CD8A, LAG3*), Cytotoxic T cell (Cytotoxic/ *CD8A*, *PRF1*), Gamma delta T cell (γδ / *TRDV1*), naïve T cell (naïve/*CCR7, TCF7, IL7R*), CD4+ naïve T cell (CD4+ naïve/ *CCR7, TCF7, IL7R, CD4*,), CD8+ naïve T cell (CD8+ naïve/, *TCF7, IL7R, CD8A*), Tcell_1 and Tcell_2 [52–54] (Figs. 5B and S4A, B*)*. With the annotated subset of T cells, the population of T cells in each stage of COPD was assessed. Alterations in T cell composition were observed across disease stages, where severe COPD T cells exhibited a pronounced expansion of CD4⁺ naïve T cells alongside a marked reduction in Th1, indicating a potential disruption in T cell differentiation and activation (Fig. 5C). To assess the functional consequences of Th1 depletion, GSEA using the Hallmark Interferon Gamma Response gene set was conducted. While bulk transcriptomic data showed increased IFN-γ–associated signaling in COPD lungs compared to healthy controls, scRNA analysis revealed a paradoxical downregulation of *IFNG* expression specifically in severe COPD, suggesting stage-specific divergence in IFN-γ signaling (Fig. 5D, E). This finding was reinforced by GSEA using the Interferon Gamma Response gene set from the Hallmark collection, which showed a significant reduction in IFN-γ response signature scores in severe COPD relative to earlier stages (Fig. 5F). Further stratified analyses demonstrated that IFN-γ expression was selectively reduced within Th1 cells in severe COPD, suggesting that Th1-associated IFN-γ activity is a feature of early disease stages but progressively diminishes with disease advancement (Fig. S4C). Consequently, comparative analyses across mild, moderate, and severe COPD T cells revealed an attenuation of Th1 transcriptional programs, with T cells from severe COPD exhibiting significantly lower Th1 activity (Fig. S4D). To further characterize the alteration in T cell dynamics, pseudotime trajectory analysis was performed using naïve T cells as the root. The trajectory revealed distinct differentiation patterns in severe COPD compared to mild and moderate COPD (Fig. 5G). Given the marked elevation of *IL6* in severe COPD, we next assessed whether sustained IL6 signaling is associated with a characteristic transcriptional imprint in T cells. Canonical *IL6*–responsive genes, including *BCL2*, *BCL3*, *FOXO1*, *NFATC2*, *PIM1*, and *SOCS1*, were preferentially upregulated in T cells from severe COPD [55–60] (Fig. 5H). In parallel, STAT3 regulon activity—a central downstream effector of IL6 signaling—was highest in severe COPD T cells, further supporting enhanced IL6 pathway activation in this context (Fig. S4E). These findings were independently validated using GSE302339, in which late-stage COPD T cells displayed significantly higher IL6 signaling scores than early-stage T cells, reinforcing the association between elevated IL6 signaling and progressive remodeling of the T cell immune landscape in severe COPD (Fig. S4F). Moreover, prior mechanistic studies have demonstrated that IL6 can directly suppress Th1 differentiation and attenuate IFN-γ signaling, providing experimental support for the mechanism proposed here in severe COPD [58]. Overall, applying machine learning to the UK Biobank proteome data and integrating transcriptional analysis of COPD patients revealed IL6 as a unique marker derived from senescent endothelial cells that regulated T cell activity in severe COPD.

## Discussion

To systematically investigate the molecular drivers of COPD severity, a multi-layered integrative analysis was conducted utilizing the UK Biobank proteome data and transcriptome of lung in COPD patients. Utilizing the UK Biobank proteome, machine–learning–based classification models identified IL6 and MMP12 as key protein biomarkers in COPD patients. Bulk and scRNA sequencing showed IL6 upregulation in severe COPD, which originated from endothelial cells characterized by senescence-associated signatures. The secreted IL6 acted on naïve T cells, leading to diminished IFN-γ signaling and a corresponding reduction in Th1 cell polarization in severe COPD. These data suggest that severe COPD senescent endothelial cells may serve as a source of elevated IL6 that downregulates IFN-γ signaling and Th1 activity. Taken together, these findings suggest IL6-mediated immune modulation in severe COPD and provide a basis for stage-specific therapeutic targeting for severe COPD.

Our analysis re-evaluates *IL6* as a prognostic biomarker and therapeutic target of severe COPD. Compared with mild COPD, severe COPD exhibits a substantially reduced cytokine response to pathogens, reflecting a marked impairment of innate immunity [61]. Machine learning analysis of the UK Biobank blood proteome identified IL6 and MMP12 as proteins upregulated in the blood serum of patients with severe COPD, with their expression levels correlating with disease severity and survival rate. However, scRNA sequencing revealed that only *IL6* exhibited stage-specific upregulation, unlike *MMP12*. IL6 is a well-established predictor of annual COPD exacerbation frequency, and IL6-neutralizing antibodies have demonstrated efficacy in alleviating key COPD symptoms such as airway mucus hypersecretion [62, 63]. Although some IL6–directed agents are FDA-approved for other diseases, none are approved for COPD. Notably, the phase III clinical trial of the IL6 receptor antagonist tocilizumab in COPD was terminated due to insufficient efficacy [64]. However, these earlier trials did not account for COPD-stage heterogeneity. Our study suggests that IL6 should be revisited as a potential therapeutic target in COPD treatment, with particular focus on severe COPD.

Our findings indicate that the upregulation of *IL6* in severe COPD is predominantly driven by endothelial cell senescence. Cellular senescence is involved in permanent cell cycle arrest and acquisition of a pro-inflammatory, tissue-destructive phenotype [65]. Senescence not only impairs the essential cellular functions, such as proliferation, but also contributes to the pathophysiology of the disease by secreting SASP into the surrounding environment [66]. Increasing evidence denotes senescence and SASP as a major pathogenic mechanism associated with COPD [21, 67]. However, the principal cell types undergoing senescence in COPD remain poorly defined. Our study addresses this gap by demonstrating that endothelial cells exhibit both enhanced senescence signatures and increased SASP production. SASP encompasses a wide range of secreted factors, including IL6, IL8, MIF, VEGF, and epiregulin [44]. Among the SASP components, IL6 is one of the major factors excreted in senescent cells and has been proven to be upregulated in endothelial cells by replication-induced senescence in vitro [68–70]. The simultaneous elevation of IL6 and SASP components in severe COPD endothelial cells suggests that IL6 upregulation in severe COPD is linked to cellular senescence and the production of SASP. Nonetheless, given the context-dependent and heterogeneous nature of SASP, further characterization of the endothelial cell SASP profile in severe COPD is necessary to fully elucidate the role of senescence and SASP in severe COPD.

To investigate the role of IL6, we examined the downstream immunological effects of IL6 signaling in severe COPD. IL6 in severe COPD was actively signaled to naïve T cells, indicating a potential role of IL6-mediated differentiation regulation. Naïve CD4⁺ T cells can differentiate into distinct effector subsets, such as Th1 and Th2, with cytokine milieu playing a decisive role in determining lineage commitment. IL6 is a well-characterized cytokine known to inhibit Th1 differentiation and suppress IFN-γ expression [58, 71]. Consistent with this mechanism, severe COPD exhibited a pronounced reduction in IFN-γ levels and Th1 abundance, supporting the hypothesis that IL6-driven signaling contributes to the decrease of Th1 and IFN-γ levels in late-stage COPD. These findings further refine current models of COPD immunopathology. Previous studies have shown that in early-stage COPD, the Th1/Th2 cytokine balance correlates with lung function indices such as FEV1% predicted and FEV1%/FVC ratio, with higher Th1 activity linked to better pulmonary function [72]. Notably, these correlations were derived from cohorts comprising only mild-to-moderate COPD patients. Our data extend this model by demonstrating that severe COPD also exhibits reduced Th1 activity. Furthermore, the accompanying reduction in IFN-γ may provide a potential mechanistic explanation for the prevalence of bacterial colonization in severe COPD compared to earlier stages, with rates reported at approximately 25% in moderate cases and over 40% in severe cases [73]. IFN-γ is a key immunoregulatory cytokine that primes immune cells such as macrophages by enhancing their responsiveness to pathogens and promoting a robust inflammatory cytokine production [74, 75]. A deficiency in IFN-γ may compromise this priming process, leading to reduced immune activation resulting in impaired pathogen clearance and an increased susceptibility to bacterial colonization in severe COPD. Collectively, these findings suggest that IL6-mediated Th1 and IFN-γ deficiency constitute a critical immunopathological mechanism linking severe COPD to decreased pulmonary function and infectious risk.

This study has several limitations. First, although severe and very severe COPD cases were consolidated into a single analytical group due to limited sample availability, this aggregation may have obscured biologically meaningful distinctions between these advanced disease stages. Second, despite the strength of integrating bulk and single-cell transcriptomic datasets—including those from the UK Biobank—the inherent heterogeneity of publicly available datasets remains a challenge. Differences in sample processing pipelines, sequencing platforms, and clinical annotation across datasets may influence transcriptional signatures and contribute to inter-cohort variability. Third, scRNAsequencing is subject to known technical constraints, including dropout effects that preferentially impact low-abundance cytokines. Consequently, sparse or absent cytokine signals should be interpreted with caution and with biological contexts. Overall, we note that while our integrative computational analysis highlights putative immune signatures in severe COPD, future studies incorporating in vitro and in vivo functional validation using primary human lung cells or relevant animal models will be necessary to reinforce the mechanistic relevance of these findings.

In this study, machine learning analysis of the UK Biobank proteome revealed IL6 as a potential marker of severity in COPD. Transcriptomic analysis further revealed that IL6 upregulation in severe COPD predominantly originates from endothelial cells exhibiting a strong senescence signature. In addition, elevated IL6 targeted T cells and reduced the Th1 population and IFN-γ production. These findings clarify the stage-specific immune alterations in severe COPD, highlighting the potential for stage-specific therapeutic strategies in treating severe COPD.

## Methods

### Utilization of UK Biobank data

The study analyzed UK Biobank data to investigate clinical patterns in individuals with COPD. Participants were identified using International Classification of Diseases, 10th Revision (ICD-10) codes J41-44 to ensure broad inclusion of COPD-related cases as detailed in Table 1. Cohort demographics and clinical characteristics are summarized in Table 2. An initial cohort of 26,033 participants was identified. After excluding individuals with missing forced expiratory volume in one second (FEV1%)/ forced vital capacity (FVC) Z-scores or normalized protein expression (NPX) values for IL6 and MMP12, a final sample of 1916 and 1573 participants was included in the analysis. All data preprocessing and visualization were conducted using R (version 4.4.2). Figures were generated with the “ggplot2” (version 3.5.1) and “ggpubr” (version 0.6.0) packages. Multivariable logistic regression analysis of factors associated with COPD was conducted using “dplyr” (version 1.1.4), “tidyverse” (version 2.0.0), and “stats” (version 4.5.2) packages.

### Machine learning analysis of UK Biobank Olink plasma proteomics for COPD classification

Analyses were implemented using the “caret” (version 7.0-1) package and the provided code [33]. Two supervised classification tasks were conducted: COPD vs control and severe vs mild COPD. For COPD vs control, data were stratified into training (2,720 COPD; 1568 control) and test (1158 COPD; 672 control) sets; for severe vs mild, training (834 mild; 154 severe), and test (120 mild; 30 severe). Preprocessing was fit on the training data—median imputation and z-score standardization via the “preProcess” function—and applied to the test set. A 20-protein panel (PC 20) was derived on the training set by repeated balanced resampling with random-forest-based recursive feature elimination and collinearity pruning, followed by rank aggregation. Using PC20 predictors, support vector machines, random forest, logistic regression, lasso (α = 1), and ridge (α = 0) were tuned with 10-fold cross-validation repeated 10 times, optimizing the area under the receiver operating characteristic curve. Variable importance and partial dependence were summarized with “randomForestSRC” (version 3.4.1). All of the analyses were computed and visualized with “pROC” (version 1.19.0.1) and “ROCR” (version 1.0-11).

### Processing bulk RNA sequencing data

To comprehensively compare the characteristics of lung tissues from COPD patients and healthy controls, the public dataset GSE239897 from the Gene Expression Omnibus (GEO) database (https://www.ncbi.nlm.nih.gov/geo/) was obtained. The analysis included lung tissue data from 43 healthy controls and 39 COPD patients.

Gene set enrichment analysis (GSEA) was performed using the GSEA software (version 4.3.2). For this, appropriate gct and cls files were prepared for each dataset and input into the program. The Hallmark gene set (h.all.v2023.2.Hs.symbols.gmt) was used as a reference. The number of permutations was set to 1000, with the gene symbols collapsed and the permutation type based on phenotype. The chip platform used was “Human_Gene_Symbol_with_Remapping_MsigDB.v2023.2.Hs.chip”. Differentially expressed genes (DEGs) were identified using the “DESeq2” (version 1.50.2) package in R

### Acquiring scRNA sequencing data

To investigate the specific characteristics associated with COPD severity, publicly available datasets from the GEO database were utilized. Lung tissue data from mild COPD patients (*n* = 7), moderate COPD patients (*n* = 7), and severe COPD patients (*n* = 3) were obtained from GSE167295, GSE173896, and GSE227691. The severity of COPD was categorized based on the Global Initiative for Chronic Obstructive Lung Disease criteria. Mild was defined as FEV1% over 80%, moderate as FEV1% between 50 and 79%, severe as FEV% 1 between 30 and 49%, and very severe as FEV1% below 30 percent. Due to the limited number of severe and very severe patients, these groups were combined and analyzed as a single severe group, resulting in three overall severity categories. Detailed patient information is provided in Table 4.

Data from a total of 17 patients were processed using the R package “Seurat” (version 5.1.0). Quality control measures were applied to exclude low-quality cells based on two criteria: the number of genes detected per cell (nFeature) and the percentage of mitochondrial genes (percent_MT). Additionally, doublets were identified and removed using the “DoubletFinder” (version 2.0.3) package. Normalization of the data was performed using the “LogNormalize” method, and the 2000 most highly variable genes were identified using the “FindvariableFeatures” function. Data integration across samples was accomplished with the “IntegrateData” function. Detailed quality control information is in Tables 5–7. As a validation dataset, a single cell dataset was utilized, GSE302339, having 62 individuals (COPD (*n* = 37) and Control (*n* = 25)) [39]. The GOLD stages were classified as GOLD 0 (*n* = 7), GOLD 1 (*n* = 7), GOLD 2 (*n* = 10), and GOLD 4 (*n* = 13). With GOLD 3 being absent, we manually divided the group into early-stage (GOLD 0, 1, 2) and late-stage (GOLD 4) and analyzed our findings.

### Utilizing scRNA sequencing data

Principal component analysis was conducted to identify the principal components relevant for downstream analysis. Based on these results, the “RunPCA” function was utilized to estimate uniform manifold approximation and projection (UMAP) coordinates using the top 30 significant principal components (Fig. S1A). A shared nearest-neighbor graph was generated through the “FindNeighbors” function applied to the UMAP coordinates. Clusters were subsequently identified by refining the shared nearest-neighbor modularity with the “FindClusters” function.

Detailed information on quality control metrics and doublet percentages is provided in Tables 5–7. The Seurat package was employed to integrate multiple Seurat objects into a single comprehensive dataset. Integration anchors were determined using the “SelectIntegrationFeatures” and “FindIntegrationAnchors” functions. Datasets were then merged with the “IntegrateData” function based on the calculated anchors. Following integration, data normalization was performed using the “NormalizeData” function. Cluster-specific marker genes were characterized by a log fold change greater than 0.25 when compared to other clusters. Finally, cluster annotation was carried out by comparing marker genes with known reference genes. Marker genes used as reference can be seen in Fig. S1B.

### Gene set enrichment analysis

GSEA software (version 4.3.2) was utilized with predefined gene sets obtained from the Molecular Signatures Database (MSigDB), including Hallmark (h.all.v2023.2.Hs.symbols.gmt) and Reactome (c2.cp.reactome.v2023.2.Hs.symbols.gmt). From the Hallmark collection, TGF_Beta_Signaling, Inflammatory response, and Interferon Gamma Signaling gene sets were used to characterize the immune landscape across COPD stages. From the Reactome database, Reactome_Senescence_Associated_Secretory_Phenotype_SASP gene set was used to assess the enrichment of senescence-associated phenotypes in endothelial cells from severe COPD samples. Manually curated gene sets, such as the Senescence_score gene sets were derived from Fridman_Senescence_Up with unexpressed genes omitted [76] (Table 8). The enrichment of each gene set was done with the “runEscape” function in the “escape (version 2. 2. 3)” package with groups set to 5000 and min. size set to 0.

### Cell–cell communication analysis

R package “CellChat” (version 1.6.1) was utilized to analyze cell-cell communication. IL6 signaling was visualized using chord diagrams generated with the “netVisual_aggregate” function. To examine the trend of IL6 signaling in endothelial cells, the “netVisual_individual” function was applied to create a circle plot.

### Volcano plot analysis

The function “FindMarker” was utilized to extract the DEGs between mild and severe endothelial cells and moderate and severe endothelial cells. The DEGs were identified and categorized into upregulated, downregulated, non-significant and senescence-related genes. These results were visualized using “GraphPad Prism” (version 9.5.1). A volcano plot was generated to effectively illustrate the distribution of these gene categories, highlighting their significance and expression levels.

### Transcription factor activity analysis

This analysis utilized the curated collection of transcription factors known as “DoRotheEA” (version 1.12.0). Human data were retrieved using the “get_dorothea” function, and to use the Weighted Mean method, the “run_wmean” function was used. This method ensures that the “wmean” is first multiplied by each target feature with its associated weight and then summed to the average of the enrichment scores. Subsequently, the data were scaled, leading to the identification of the top transcription factors with variable means across the clusters.

### T cell differentiation trajectory mapping

Differentiation trajectories of T cells across COPD severities were inferred using the R package “slingshot” (version 2.14.0). For each disease group, cells were subset based on classification, and an object was initialized with a placeholder count matrix. The “slingshot” function was applied using UMAP coordinates as “reducedDim” to estimate developmental lineages and pseudotime. Lineage structures were visualized through the “SlingshotDataSet” function. Trajectory inference was performed independently for mild, moderate, and severe COPD groups using the same preprocessing and analysis steps.

### Xenium data processing and cell type annotation

Xenium spatial transcriptomics data were processed using “Seurat” (version 5.3.1) and analyzed in conjunction with “spacexr” (version 2.2.1). The publicly available dataset GSE313006 was utilized, comprising 4 tissue microarray cores from patients with COPD. Data import was performed using the “LoadXenium” function, followed by standard preprocessing steps. Only cells with at least one detected transcript (nCount_Xenium > 0) were retained for downstream analysis to remove empty or low-quality segments.

Gene expression values were normalized and variance-stabilized using “SCTransform” applied to the xenium assay. Principal component analysis was then performed using the full set of detected genes with “RunPCA”, and the top 30 principal components were retained for downstream dimensionality reduction and clustering. “RunUMAP” was used to visualize the data in two dimensions based on these components. A shared nearest-neighbor graph was constructed by using “FindNeighbors” using the principal component analysis reduction, followed by “FindClusters” with a resolution parameter of 0.5. Spatial visualization of transcript distributions was performed using “ImageDimPlot”, overlaying detected molecules onto the tissue field of view with molecule transparency adjusted to facilitate visualization of spatial patterns.

To infer cell-type identities, the “create.RCTD” and “run. RCTD” function was used. Raw count matrices, subclass labels, and per-cell UMI counts were extracted from the reference dataset and used to construct a reference object for robust cell type decomposition. Cell-type predictions were obtained from the primary annotation output of the robust cell type decomposition results, and predicted labels were subsequently integrated into the Xenium Seurat object as metadata.

### COPD cell atlas

The COPD Cell Atlas is a publicly available single-cell transcriptomic resource and can be accessed through the GEO database under accession number GSE136831, as well as via the dedicated COPD Cell Atlas web portal www.copdcellatlas.com [40]. Data were queried using the “Gene Explorer” module of the COPD Cell Atlas web interface. For cell-type–specific analysis, the endothelial cell population was selected, and gene expression values were extracted using the “average per subject” aggregation setting. The extracted data were subsequently used for downstream analyses and visualization as described below.

## Supplementary information

Supplementary material

## Data Availability

Plasma proteomic data used in this study were obtained from the UK Biobank under approved application number 177007. The bulk RNA sequencing dataset analyzed in this study was obtained from the GEO under the accession number GSE239897. Additionally, scRNA sequencing datasets were acquired from the GEO database, specifically under the accession numbers GSE227691, GSE173896, GSE167295 and GSE302339. Spatial transcriptomic Xenium data were also acquired from the GEO database, derived from GSE313006. COPD Cell Atlas data were derived from www.copdcellatlas.com.
