# Supplementary material for: Endothelial cell senescence shapes T cell activity in late-stage of chronic obstructive pulmonary disease
Source: Cell Death Discov. 2026 Mar 20;12:160. doi: 10.1038/s41420-026-03020-2 (PMC13039841; doi:10.1038/s41420-026-03020-2)
Supplement: Supplementary file 1 — Supplementary material [file 41420_2026_3020_MOESM1_ESM.docx]

**Fig. S1 scRNA sequencing data processing**


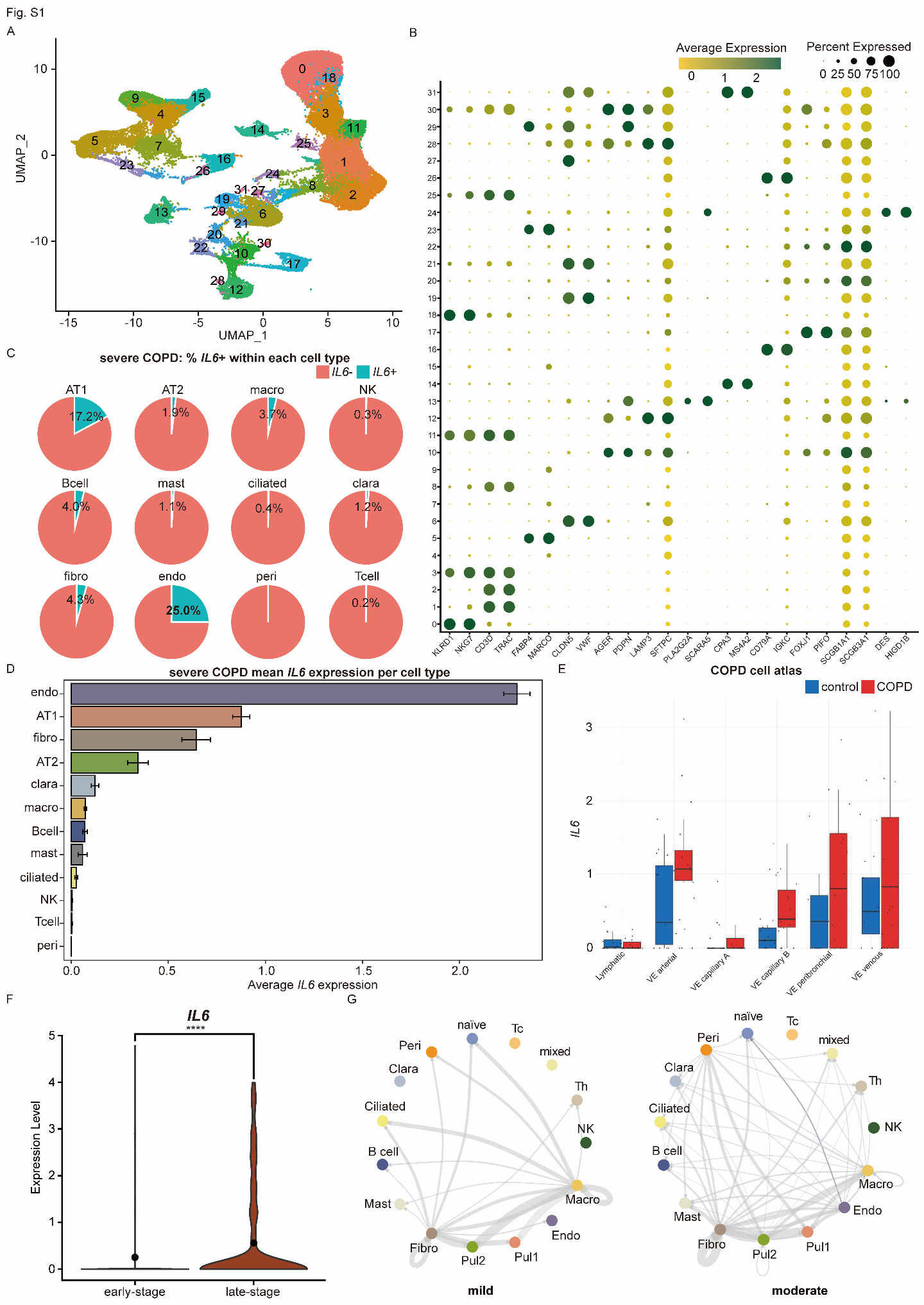


**A** Dim plot showing approximately 32 clusters. **B** Dot plot showing marker gene reference that was used to annotate the cells for downstream analysis. **C** Pie charts showing the proportion of IL6-expressing cells in severe COPD for each cell type, highlighting endothelial cells as the predominant IL6-positive population. **D** Bar plot showing the average IL6 expression across cell types in severe COPD with endothelial cells exhibiting the highest average expression levels**. E** Box plot of endothelial cells in COPD Cell Atlas reveals a pronounced elevation of IL6 expression in COPD samples relative to controls, underscoring IL6 as a cytokine selectively upregulated in COPD. **F** Violin plot of COPD dataset GSE302339 demonstrate that endothelial cells in late-stage COPD expresses higher IL6 than that of early-stage COPD endothelial cells *(*** p < 0.001)*. **G** Chord diagram shows IL6 signaling between cell in mild COPD (left) and in moderate COPD (right); Natural killer cell (NK cell); Mast cell (Mast); Macrophage (Macro); B cell (B cell); Pericyte (Peri); Endothelial cell (Endo); Fibroblast (Fibro); Clara cell (Clara); Pulmonary alveolar type I (AT1); Pulmonary alveolar type II (AT2); Ciliated cell (Ciliated); Tcell (T cells)

**Fig. S2 TGF-β signaling of endothelial cells in mild, moderate and severe COPD**

**
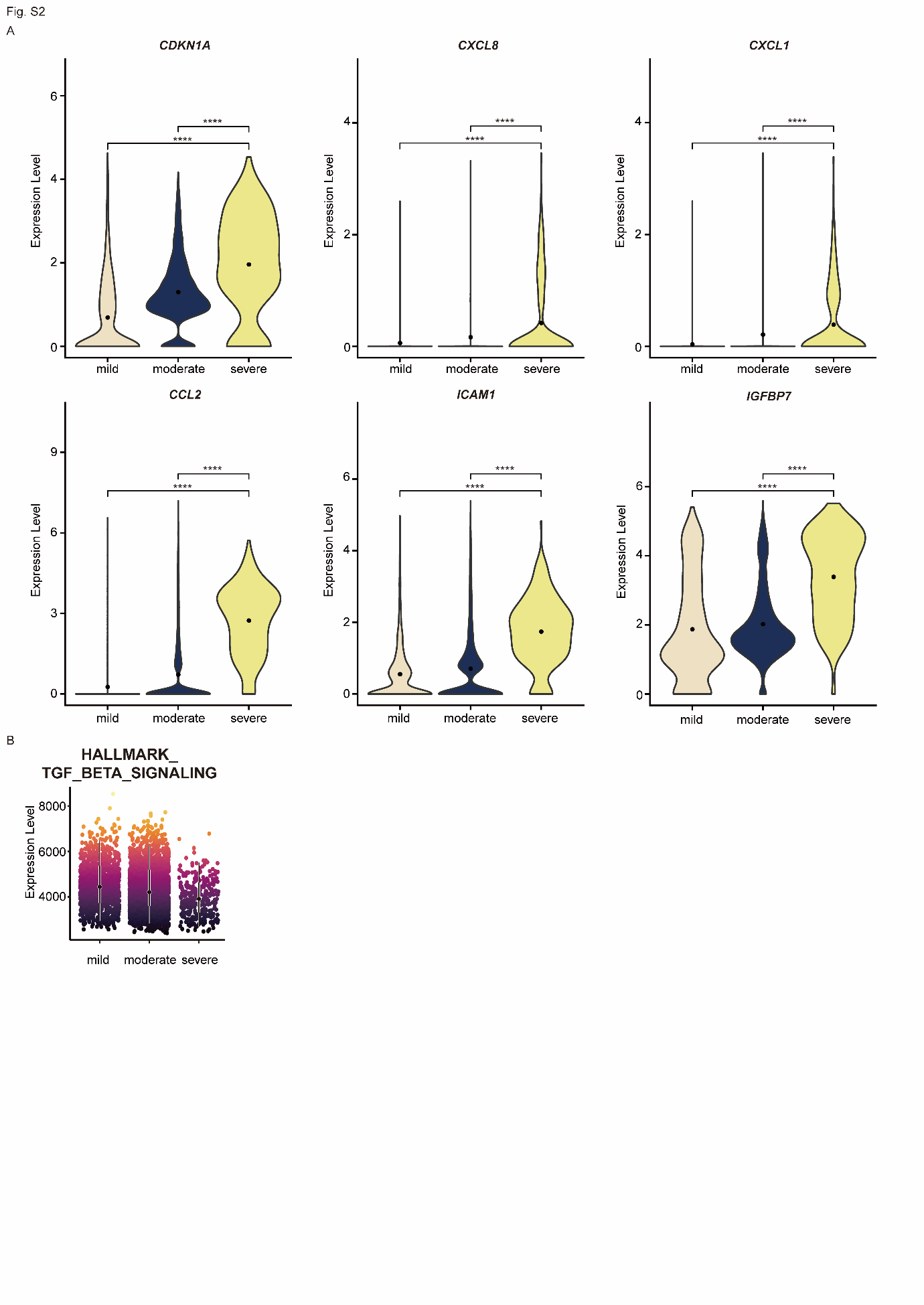
**

**A** Violin plots of canonical senescence markers (*CDKN1A*, *CXCL8*, *CXCL1*, *CCL2*, *ICAM1*, and *IGFBP7*) reveal elevated expression in endothelial cells from severe COPD, providing transcriptomic evidence consistent with heightened endothelial senescence in severe COPD (**** p < 0.0001). **B** Geyser plot of Hallmark TGF-β signaling showing the reduction of TGF-β signaling in endothelial cells in severe COPD compared to endothelial cells in mild and moderate COPD.

**Fig. S3 Xenium data processing and analysis for IL6 and endothelial cell senescence**

**
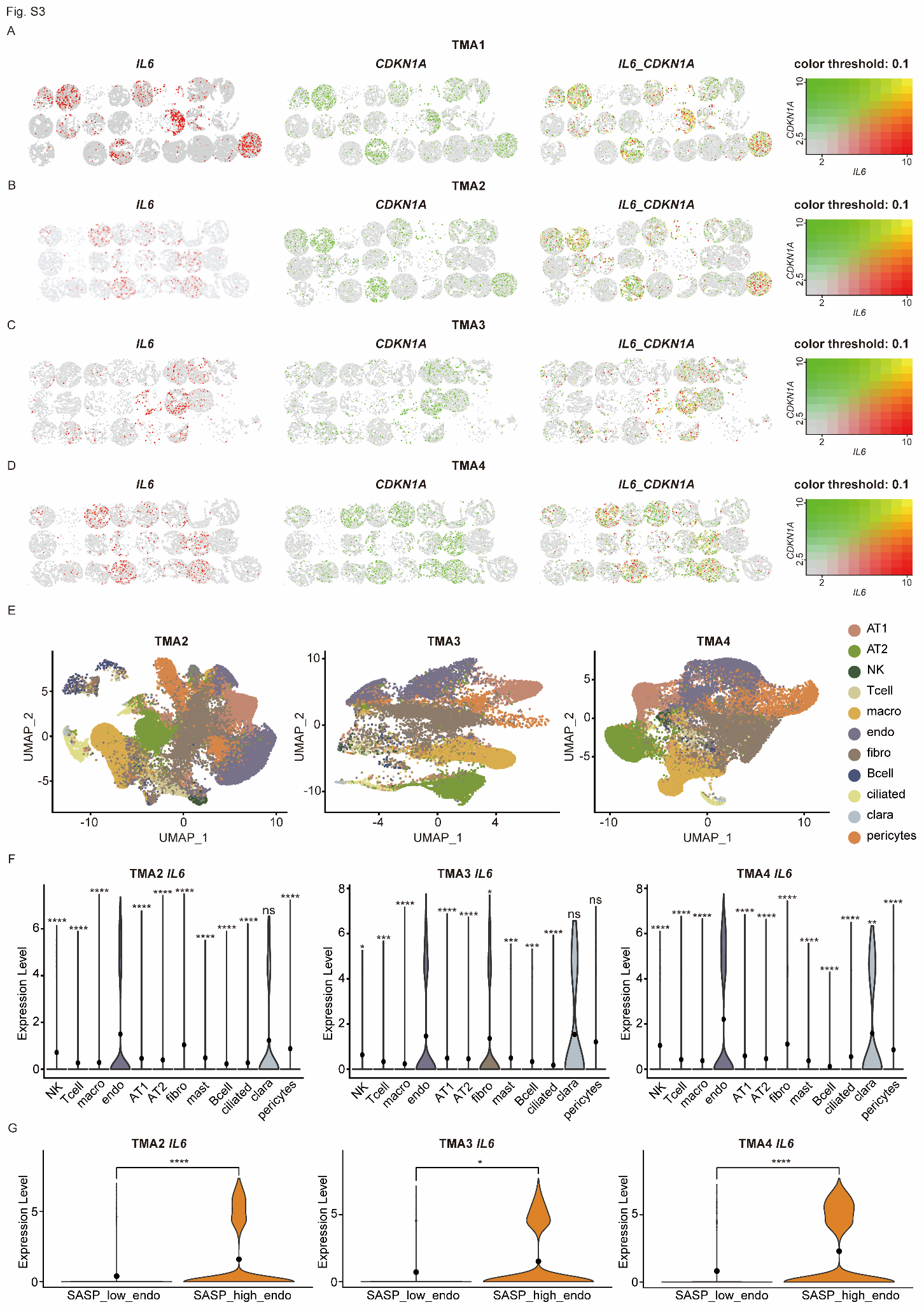
**

**A-D** Spatial imaging plots illustrate the colocalization of IL6 and CDKN1A in TMA1 (A), TMA2 (B), TMA3 (C), and TMA4 COPD samples (D). **E** Dim plot showing the annotated cell types of TMA2 (left), TMA3 (middle), and TMA4 (right) samples derived from the GSE313006. **F** Violin plots demonstrating significantly elevated IL6 expression in endothelial cells compared with other cell types in TMA2 (left), TMA3 (middle), and TMA4 (right) (ns=not significant * p < 0.1, ** p < 0.01, *** p < 0.001, **** p < 0.0001). **G** Violin plots showing that senescence-high endothelial cells exhibit markedly increased IL6 expression relative to senescence-low endothelial cells (** p < 0.1,* **** p < 0.0001); Senescence associated secretory phenotype (SASP)

**Fig. S4 scRNA sequencing data processing of T cells**


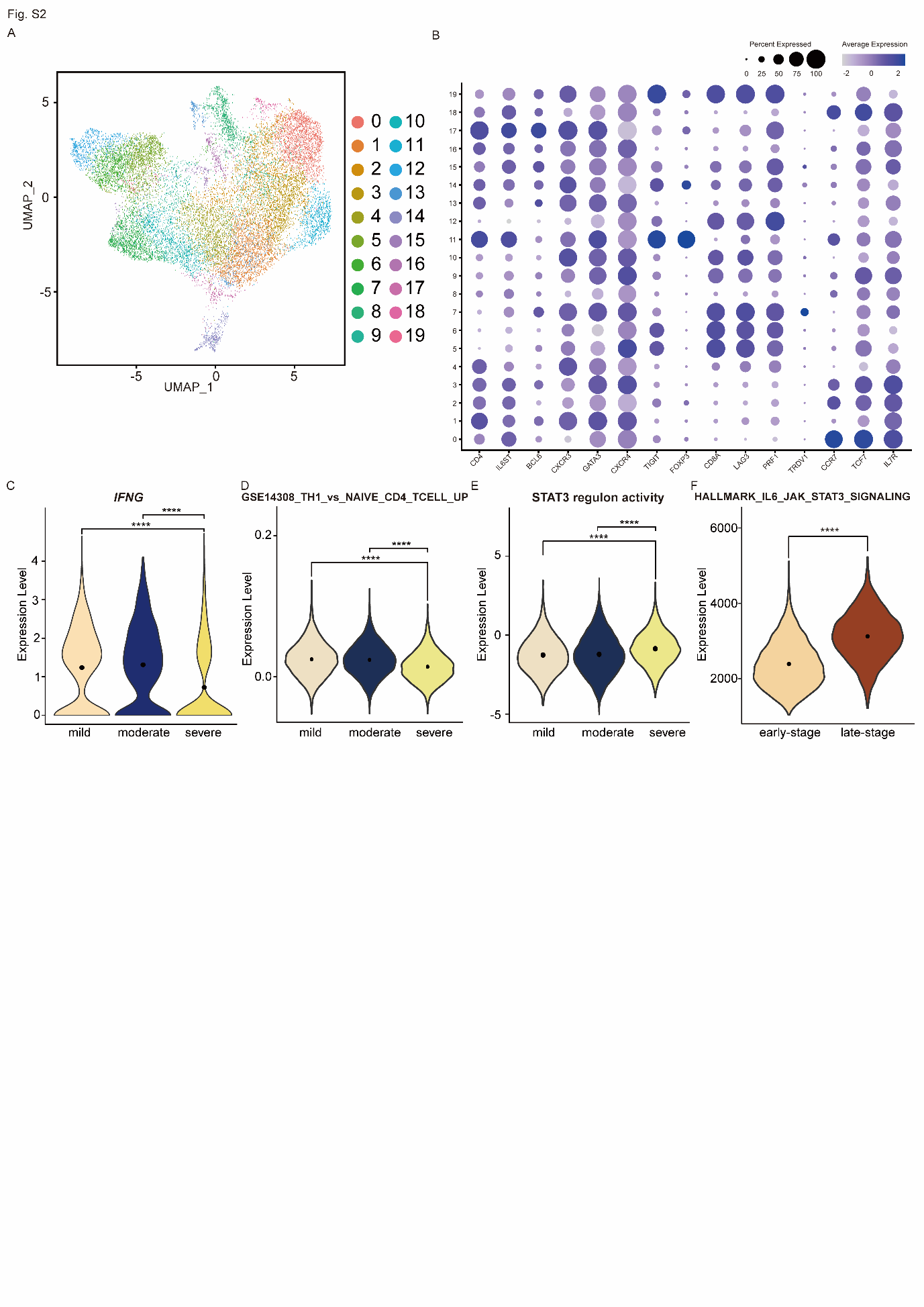


**A** Dim plot showing approximately 20 clusters. **B** Dot plot showing marker gene reference that was used to annotate subset of T cells for downstream analysis. **C** Violin plot showing decrease of IFN-γ expression in Th1 in severe COPD compared to mild and moderate COPD (**** p < 0.0001). **D** Violin plot showing reduced Th1 transcriptional activity in T cells from severe COPD, indicating impaired Th1 immunity (**** p < 0.0001). **E** Violin plot illustrating increased STAT3 regulon activity in T cells from severe COPD relative to mild and moderate cases, indicating upregulated IL6– mediated signaling (**** p < 0.0001). **F** Violin plot from the independent dataset GSE302339 show that T cells in late-stage COPD exhibit markedly higher IL6-JAK-STAT3 pathway compared with early COPD states (**** p < 0.0001)
